# Three-dimensional tumor spheroids for in vitro analysis of bacteria as gene delivery vectors in tumor therapy

**DOI:** 10.1186/s12934-015-0383-5

**Published:** 2015-12-12

**Authors:** Annika Osswald, Zhongke Sun, Verena Grimm, Grace Ampem, Karin Riegel, Astrid M. Westendorf, Wolfgang Sommergruber, Kerstin Otte, Peter Dürre, Christian U. Riedel

**Affiliations:** Institute of Microbiology and Biotechnology, University of Ulm, 89069 Ulm, Germany; College of Life Sciences and Agriculture, Zhoukou Normal University, Chuanhui District, Zhoukou, 466001 People’s Republic of China; Institute of Medical Microbiology, University Hospital Essen, University Duisburg-Essen, Essen, Germany; Department of Lead Discovery, Boehringer Ingelheim RCV GmbH & Co KG, 1121 Vienna, Austria; Institute of Applied Biotechnology, University of Applied Sciences Biberach, 88400 Biberach, Germany

**Keywords:** Bacteria, Gene delivery vector, Tumor targeting, Prodrug converting enzyme, Tumor spheroids

## Abstract

**Background:**

Several studies in animal models demonstrated that obligate and facultative anaerobic bacteria of the genera *Bifidobacterium*, *Salmonella*, or *Clostridium* specifically colonize solid tumors. Consequently, these and other bacteria are discussed as live vectors to deliver therapeutic genes to inhibit tumor growth. Therapeutic approaches for cancer treatment using anaerobic bacteria have been investigated in different mouse models. In the present study, solid three-dimensional (3D) multicellular tumor spheroids (MCTS) of the colorectal adenocarcinoma cell line HT-29 were generated and tested for their potential to study prodrug-converting enzyme therapies using bacterial vectors in vitro.

**Results:**

HT-29 MCTS resembled solid tumors displaying all relevant features with an outer zone of proliferating cells and hypoxic and apoptotic regions in the core. Upon incubation with HT-29 MCTS, *Bifidobacterium bifidum* S17 and *Salmonella typhimurium* YB1 selectively localized, survived and replicated in hypoxic areas inside MCTS. Furthermore, spores of the obligate anaerobe *Clostridium sporogenes* germinated in these hypoxic areas. To further evaluate the potential of MCTS to investigate therapeutic approaches using bacteria as gene delivery vectors, recombinant bifidobacteria expressing prodrug-converting enzymes were used. Expression of a secreted cytosine deaminase in combination with 5-fluorocytosine had no effect on growth of MCTS due to an intrinsic resistance of HT-29 cells to 5-fluorouracil, i.e. the converted drug. However, a combination of the prodrug CB1954 and a strain expressing a secreted chromate reductase effectively inhibited MCTS growth.

**Conclusions:**

Collectively, the presented results indicate that MCTS are a suitable and reliable model to investigate live bacteria as gene delivery vectors for cancer therapy in vitro.

**Electronic supplementary material:**

The online version of this article (doi:10.1186/s12934-015-0383-5) contains supplementary material, which is available to authorized users.

## Background

Solid tumors are characterized by hypoxic and necrotic areas, which are the result of high metabolic activity of tumor tissue and concomitant lack of oxygen supply due to insufficient vasculature [1, 2]. Hypoxic areas of solid tumors have an increased resistance to chemotherapy and radiation compared to better oxygenated tumor tissue [3–7]. This has fuelled the search for alternative or supplementary therapeutic strategies. One promising approach is the use of bacterial vectors for expression of therapeutic genes directly in tumor tissue. Different bacterial species including *Escherichia coli* [8, 9] and *Listeria monocytogenes* [10, 11] were shown to selectively colonize tumors in various animal models. The most frequently investigated bacterial gene delivery systems to target solid tumors are, however, facultative or obligate anaerobes of the genera *Salmonella*, *Clostridium*, and *Bifidobacterium* (reviewed in [12–15]).

Bifidobacteria are Gram-positive, obligate anaerobic bacteria of the normal human intestinal microbiota. Several studies in animal models have shown that bifidobacteria selectively colonize and replicate in solid tumors following oral, intravenous, or intratumoral application [16, 17]. Due to their non-pathogenic nature and broad use as probiotics, bifidobacteria are promising candidates as live vectors for delivery and expression of therapeutic genes to inhibit tumor growth. However, most bifidobacteria are highly resistant to genetic manipulation and only a very limited number of strains have been modified to express genes relevant for tumor therapy [18–20].

Another group of Gram-positive, strictly anaerobic bacteria widely used for tumor targeting strategies are spore-forming *Clostridium* sp. [21, 22]. In early studies, clostridia were mainly used as tumor therapeutics based on their oncolytic activity (reviewed in [14, 22]). However, their oncolytic effect was limited to large tumors with extensive and strictly hypoxic areas, tumor regression was incomplete and experimental animals died from clostridial toxins [14]. Consequently, this problem was addressed by generation of attenuated or non-pathogenic strains [23–25]. A major advantage of clostridia is that these microorganisms are able to form spores, which are immunologically inert and can be administered safely by intravenous injection [22, 26]. Spores of various *Clostridium* sp. were shown to selectively germinate in tumors and vegetative cells are non-viable in other, more oxygenated tissues [22]. These features make spores an attractive alternative for administration of bacterial gene vectors to tumor patients. A clinical phase I safety study utilizing *Clostridium novyi*-NT spores has been performed in patients with various treatment-refractory solid tumor malignancies [27]. The non-toxic *C. novyi*-NT strain was generated by inactivation of the phage carrying the lethal *C. novyi* toxin [25]. In animal experiments, a single intravenous injection of *C. novyi*-NT spores resulted in efficient colonization of tumors and caused tumor regression. Moreover, tumors did not recur in approximately 30 % of the animals [25, 28].

Similarly, facultative anaerobic *Salmonella* sp. were shown to colonize solid tumors [29, 30]. Various recombinant *Salmonella typhimurium* strains have been generated for different therapeutic strategies, e.g. expression of PCEs, immunomodulatory molecules or bacterial toxins (reviewed in [29, 30]). Additionally, several attempts were made to improve tumor colonization of *S. typhimurium* and to reduce effects of this human pathogen on normal tissues [31]. For example, deletion of *msbB*, whose gene product is involved in myristoylation of lipid A, results in attenuated virulence and a reduced inflammatory response [32]. Tumor-specificity was improved by generation of mutant strains with metabolic defects that can be complemented by nutrients specifically available in tumors. To this end, genes such as *aroA* and *purI*, which encode for crucial steps in purine and amino acid biosynthesis, were targets for the generation of auxotrophic mutants of *S. typhimurium* [33–35].

Based on the attenuated vaccine strain *S. typhimurium* SL7207, which carries an inactivated *aroA* gene [33], the “obligate” anaerobic *S. typhimurium* YB1 was generated [36]. *S. typhimurium* YB1 expresses an RNA molecule complementary to the *asd* mRNA exclusively under aerobic conditions. This silences *asd* expression by an anti-sense RNA mechanism during aerobic growth. The *asd* gene encodes for an enzyme required for biosynthesis of diaminopimelic acid (DAP), which is an essential component of the bacterial cell wall. In consequence, *S. typhimurium* YB1 is auxotrophic for DAP under aerobic conditions but is able to grow in the absence of DAP under anaerobic conditions when the anti-sense RNA is not expressed. The result is an attenuated strain that shows improved colonization and survival in hypoxic areas of tumors but not in other, more oxygenated organs of mice [36].

A widely used in vitro model system in preclinical cancer research are three-dimensional (3D) spherical cell aggregates, so-called multicellular tumor spheroids (MCTS), which are formed by a wide range of tumor cell lines when cultured under appropriate conditions [37, 38]. These MCTS display characteristic features of solid tumors including different zones of proliferating, apoptotic, and necrotic cells and an oxygen gradient with hypoxic areas in the core [3, 37–39]. Besides the physiological parameters, MCTS also more closely resemble the global expression profiles of tumor biopsies than classical 2D cell cultures [40, 41]. Based on these properties, MCTS grown in vitro are increasingly used for drug discovery and pharmacokinetic and pharmacodynamic studies [37–39, 42].

The objective of the presented study was to evaluate the potential of MCTS as an in vitro model system for preclinical investigation of therapeutic strategies to treat cancer using anaerobic bacteria as gene delivery vectors.

## Results

### Characterization of MCTS of the colorectal adenocarcinoma cell line HT-29

Previous studies have shown that different bacteria are able to selectively colonize subcutaneous tumors in mice [16, 17]. In order to investigate bacterial tumor targeting in vitro, MCTS of the colorectal cancer cell line HT-29 were established as a 3D model for solid tumors. For the generation of MCTS, HT-29 cells were grown in ultra-low attachment cell culture plates. Over a 14-day period, MCTS steadily increased in size (data not shown) and reached a diameter of 500–1000 µm. HT-29 MCTS displayed an intense purple hematoxylin staining in the outer layers of the MCTS, which gradually fainted into a pink eosin staining towards the core (Additional file 1: Figure S1A). The inner core of MCTS completely lacked fixable structures. Moreover, HT-29 MCTS were characterized by hypoxic areas inside MCTS starting approximately 100 µm below the outer surface and apoptotic cells appeared another 10–20 µm further towards the core (Additional file 1: Figure S1B). Proliferating tumor cells were observed exclusively on the outermost 100 µm in the area where actin staining indicates an intact cytoskeleton (Additional file 1: Figure S1C).

### Localization and survival of *B. bifidum* S17 in MCTS

**Fig. 1:**
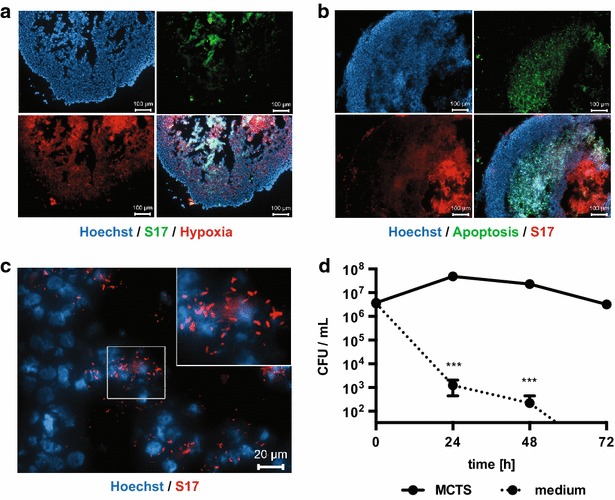
Localization and survival of *B. bifidum* S17 in MCTS. HT-29 MCTS were incubated with *B. bifidum* S17/pVG-GFP (**a** S17, *green*) or *B. bifidum* S17/pVG-mCherry (**b**, **c** S17, *red*) for 48 h. Cryosections of MCTS were stained for hypoxia (**a** Hypoxyprobe-Red549, *red*) or apoptosis (**b** TUNEL, *green*). Nuclei were stained with Hoechst. Images were acquired with a ×10 (in **a**, **b** *scale bars* 100 µm; all three fluorescence channels are shown plus the merged image in the *lower right panel*) or ×40 (in **c** *scale bar* 20 µm) objective. The *inset* in the *upper right corner* of **c** is a digital zoom on the image part marked with a *white box*. **d** Viable bacteria of *B. bifidum* S17 WT in HT-29 MCTS were determined at different time points after inoculation. As controls (medium), bacteria were incubated under identical conditions in the absence of MCTS. For each strain, time-point and condition, six tumors were infected and combined for preparation of tumor lysates. Values are colony-forming units (CFU) per mL lysate and are mean ± standard error of the mean (SEM) of three tumor lysates infected with independent bacterial cultures. Statistical analysis was performed for each time-point using Student’s *t* test (***P < 0.001)

Since MCTS display hypoxic areas similar to those observed in tumors in vivo, the ability of obligate anaerobic bifidobacteria to colonize MCTS in vitro was tested. *B. bifidum* S17 or fluorescent derivatives were incubated with MCTS for 48 h. In cryosections of these MCTS, *B. bifidum* S17 localized predominantly in the core of MCTS and this signal co-localized with hypoxic areas (Fig. 1a) and the necrotic regions of MCTS inside the apoptotic areas (Fig. 1b). In higher magnification images, individual, rod-shaped, red-fluorescent *B. bifidum* S17/pVG-mCherry could be visualized around Hoechst-stained cell nuclei indicating a close association of bacteria with tumor cells (Fig. 1c).

In order to check if *B. bifidum* S17 is able to reach the core of MCTS in a viable state and survive in this niche for prolonged periods, experiments were repeated and viable bacteria inside MCTS were quantified for up to 72 h following infection. Indeed, the number of viable *B. bifidum* S17 remained more or less constant throughout the experiment (Fig. 1d). By contrast, in plain culture media, i.e. in the absence of MCTS, viable *B. bifidum* S17 counts dropped by more than 4 orders of magnitude within the first 24 h and were below the detection limit after 72 h. Control experiments were performed by incubation of *B. bifidum* S17 with monolayers of HT-29 cells. As observed for plain culture media, viable counts dropped below the detection limit within 72 h (data not shown) demonstrating that presence of HT-29 cells alone is not sufficient but instead formation of MCTS is required to maintain viability.

### Colonization of HT-29 MCTS by other tumor targeting bacteria

**Fig. 2:**
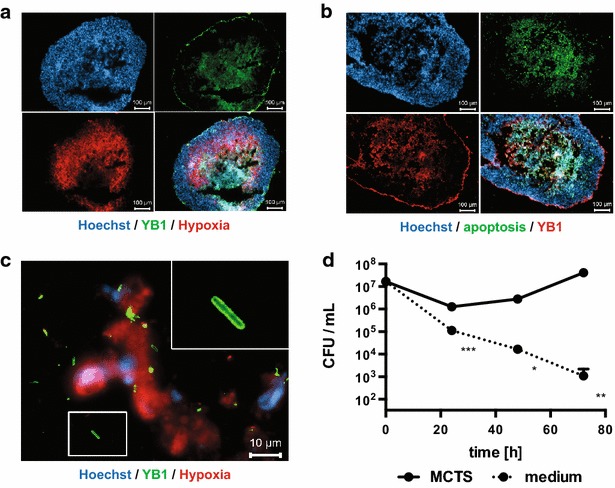
Localization and survival of *S. typhimurium* YB1 in MCTS. HT-29 MCTS were incubated with *S. typhimurium* YB1 (YB1, *green*). After 48 h, MCTS cryosections were stained for *S. typhimurium* (**a**, **c** Alexa Fluor^®^ 488-conjugated secondary antibody, *green*; **b** Alexa Fluor^®^ 555-conjugated secondary antibody, *red*) and hypoxia (**a**, **c** Hypoxyprobe, *red*) or apoptosis (**b** TUNEL, *green*). Nuclei were stained with Hoechst (*blue*). Images were acquired with a ×10 (**a**, **b** *scale bars* 100 µm; all three fluorescence channels are shown plus the merged image in the *lower right panel*) or ×100 (in **c** *scale bar* 10 µm) objective. The image in **c** is acquired at a location inside the hypoxic area of an MCTS. The *inset* in the *upper right corner* of **c** is a digital zoom on the image part marked with a *white box*. **d** Viable bacteria of *S. typhimurium* YB1 in HT-29 MCTS at different time points after inoculation. As controls (medium), bacteria were incubated under identical conditions in the absence of MCTS. For each strain, time-point and condition, six tumours were infected and combined for preparation of tumour lysates. Values are colony-forming units (CFU) per mL lysate and are mean ± SEM of three tumour lysates infected with independent bacterial cultures (in some cases, *error bars* are smaller that graph symbols). Statistical analysis was performed using Student’s *t* test (*P < 0.05; **P < 0.01; ***P < 0.001)

*Salmonella* sp. are the most frequently used bacteria in tumor targeting studies. One example is *S. typhimurium* YB1, a strain that was rendered obligate anaerobic to improve selective localization and survival in hypoxic tumor tissue [36]. Following co-incubation, *S. typhimurium* YB1 was mainly present in the core of MCTS although some bacteria were also detected on the outer surface of the MCTS (Fig. 2a). Moreover, bacteria were present mainly in areas that also stained positive for hypoxia and apoptosis (Fig. 2a–c). Experiments to quantify viable bacteria in MCTS showed that *S. typhimurium* YB1 is able to survive inside tumor spheroids for at least 72 h (Fig. 2d). In fact, after a slight decrease during the first 24 h, viable *S. typhimurium* YB1 increased tenfold over the next 48 h indicating replication of *S. typhimurium* YB1 in MCTS. Over the same period, the number of viable bacteria in controls (i.e. bacteria in medium without MCTS) decreased by about 4 orders of magnitude.

**Fig. 3:**
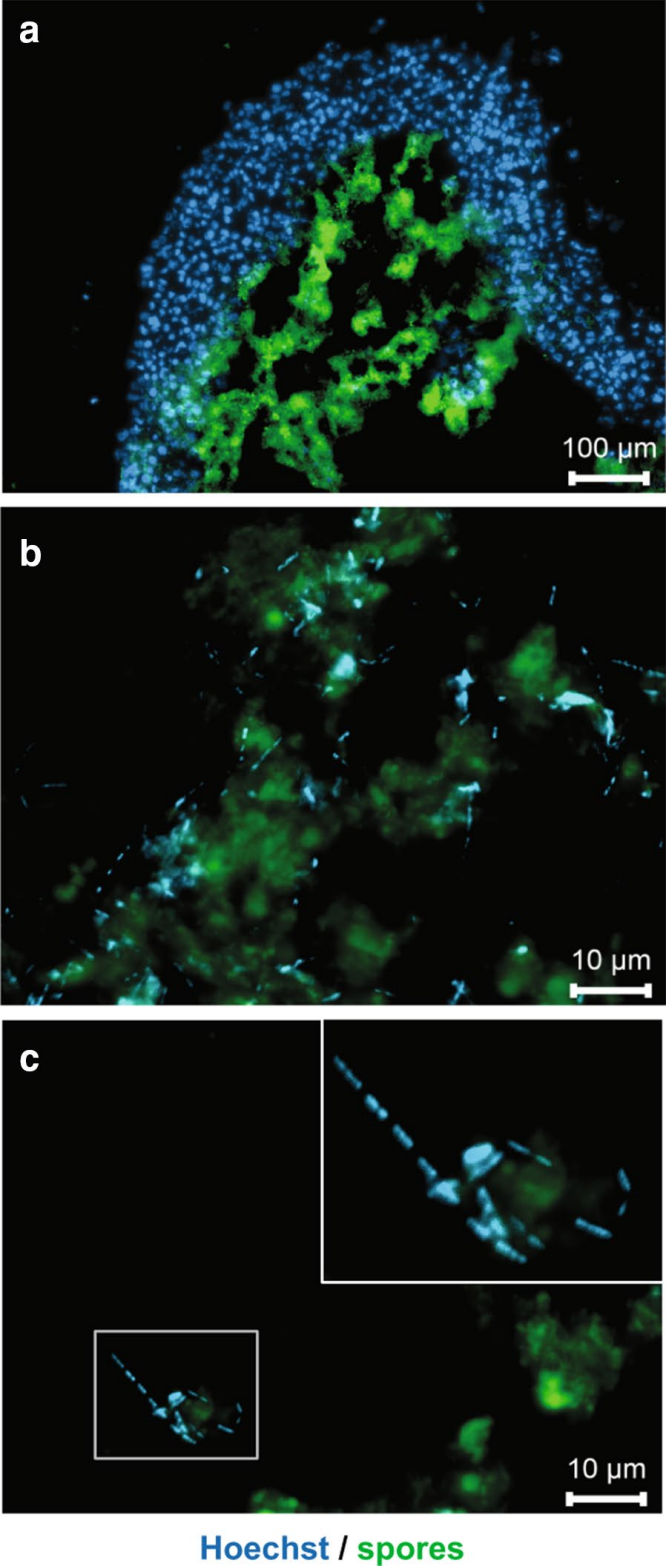
Germination of *C. sporogenes* spores in MCTS. HT-29 MCTS were infected with 1 × 10^3^ spores and incubated for 7 days. Cryosections of MCTS were then stained for spores (FITC-conjugated antiserum, *green*). Nuclei and DNA of vegetative bacterial cells were stained with Hoechst (*blue*). Images were acquired with a ×10 (in **a** *scale bar* 100 µm), or ×100 (in **b**, **c** *scale bars* 10 µm) objective. Higher magnification images in **b**, **c** are acquired at locations inside the hypoxic area of MCTS. The *inset* in the *upper right corner* of **c** is a digital zoom on the image part marked with a *white box*. Images are composite of both fluorescence channels and one representative image of three independent experiments is shown

Another group of bacteria frequently used as gene delivery vector in cancer therapy are spore-forming clostridia [22]. In order to test the utility of MCTS to study tumor targeting using clostridial spores, MCTS were incubated for 7 days with a pure spore preparation of *C. sporogenes* NCIMB 10696. Clostridial spores accumulated exclusively inside MCTS starting about 100 µm underneath the outer surface (Fig. 3a), which correlates with the beginning of hypoxia (Additional file 1: Figure S1B). Upon closer examination, structures that stained positive for Hoechst were observed in areas of stained spores (Fig. 3b). These structures clearly differed morphologically from cell nuclei and had the size and shape of bacilli, i.e. the vegetative form of *C. sporogenes* (Fig. 3c) suggesting germination of spores.

### Generation of HT-29/GFP MCTS

In order to test potential therapeutic strategies using bacteria as gene delivery vectors, fluorescent MCTS were generated. For this purpose, HT-29 cells were stably transfected with pEGFP-N3 (Clontech Laboratories, USA; Additional file 2: Materials S2). HT-29/EGFP4, a clone exhibiting stable, bright fluorescence, absence of EGFP-negative cells (Additional file 3: Figure S2A) and identical growth characteristics as the parental cell line (data not shown), was selected for further experiments. After 14 days of incubation, MCTS of HT-29/EGFP4 cells had the same size as those of the parental cell line but showed a uniform green fluorescent label throughout the MCTS (Additional file 3: Figure S2B). To test if strategies to inhibit growth of MCTS can be monitored using this system, mature HT-29/EGFP4 MCTS were treated with staurosporine. Fluorescence intensity of untreated HT-29/EGFP4 MCTS increased by 70–80 % over a 96 h period. By contrast, HT-29/EGFP4 MCTS treated with 30 or 300 nM staurosporine showed no increase in EGFP fluorescence (Additional file 3: Figure S2C) indicating efficient inhibition of MCTS growth. This suggests that HT-29/EGFP4 MCTS are a valid model to study therapeutic approaches to inhibit tumor growth.

### Evaluation of PCE strategies in HT-29/GFP MCTS

**Fig. 4:**
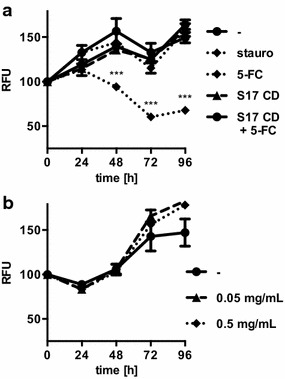
Effect of *B. bifidum* S17/pAO-S0_CD and 5-FC on HT-29/EGFP4 MCTS. **a** HT-29/EGFP4 MCTS were incubated with *B.* *bifidum* S17/pAO-S0_CD (S17 CD) alone or in combination with 0.25 mg/mL 5-FC up to for 96 h. As controls, MCTS were treated with 200 nM staurosporine (stauro), 5-FC, or medium alone (-). **b** HT-29/EGFP4 MCTS were grown in the presence and absence of 0.5 or 0.05 mg/mL 5-FU. All MCTS were analyzed for growth by fluorescence measurements for 96 h. Fluorescence intensities were normalized to give 100 % for each MCTS at the start of the experiment (t = 0 h). Values are relative fluorescent units (RFU) per MCTS and are mean ± SEM of three MCTS. Results of one representative of three independent experiments are shown. Statistical analysis was performed using two-way ANOVA with Bonferroni post-test correction for multiple comparisons (***difference to untreated MCTS significant with adjusted *P* < 0.001, all other comparisons not significant)

In a previous study, we generated *B. bifidum* S17/pAO-S0_CD, a strain expressing a secreted derivative of the *E. coli* cytosine deaminase [43]. This bacterial enzyme is not present in mammalian cells and converts the non-toxic prodrug 5-FC to the potent cytostatic compound 5-FU, which inhibits DNA synthesis. The potential of *B. bifidum* S17/pAO-S0_CD in combination with 5-FC to inhibit tumor growth in vitro was analyzed using HT-29/EGFP4 MCTS (Fig. 4a). Fluorescence of untreated MCTS increased by approx. 60 % over the 96 h monitored and staurosporine-treated control MCTS showed significantly decreased EGFP intensity. Neither *B. bifidum* S17/pAO-S0_CD, nor 5-FC, or the combination of both, resulted in a reduction of the fluorescence intensity below control levels.

One possible explanation for the lack of an effect on MCTS by the combination of *B. bifidum* S17/pAO-S0_CD and 5-FC could be an intrinsic resistance of the HT-29 cell line to 5-FU treatment, as reported for different colon cancer cells [44, 45]. To test this hypothesis, the pure compound 5-FU was added to HT29/EGFP4 MCTS. Interestingly, none of the tested concentrations of 5-FU was able to inhibit the increase in EGFP intensity during MCTS growth, indicating that the clone of HT-29 cells used to generate MCTS was resistant to 5-FU (Fig. 4b).

**Fig. 5:**
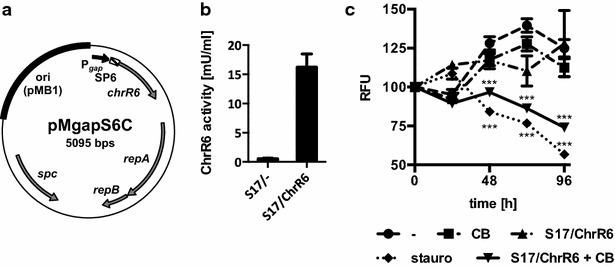
Effect of *B. bifidum* S17/pMgapS6C and CB1954 on HT-29/EGFP4 MCTS. **a** Vector map of pMgapS6C for expression of a secreted chromate reductase in bifidobacteria. **b** Chromate reductase activity in the supernatants of *B. bifidum* S17/pMgapS6C (S17/ChrR6) or the empty vector control strain *B. bifidum* S17/pMDY23-P_gap_ (S17/-). **c** HT-29/EGFP4 MCTS were incubated with S17/ChrR6 alone or in combination with 100 µM CB1954 (CB) for up to 96 h. As controls, MCTS were treated with 200 nM staurosporine (stauro), CB1954 (CB) or medium alone (-). Fluorescence intensities were normalized to give 100 % for each MCTS at the start of the experiment (t = 0 h). Values are relative fluorescent units (RFU) per MCTS and are mean ± SEM of three MCTS. Results of one representative of three independent experiments are shown. Statistical analysis was performed using two-way ANOVA with Bonferroni post-test correction for multiple comparisons (***difference to untreated MCTS significant with adjusted *P* < 0.001, all other comparisons not significant)

To test a second PCE strategy, pMgapS6C (Fig. 5a) was generated and transformed into *B. bifidum* S17. The recombinant strain *B. bifidum* S17/pMgapS6C expresses a secreted chromate reductase. This enzyme belongs to the family of nitroreductases and converts the prodrug CB1954 to hydroxylamines, which intercalate into DNA and efficiently inhibit tumor cell growth [46]. Culture supernatants of *B. bifidum* S17/pMgapS6C contained highly increased nitroreductase activity compared to the control strain (Fig. 5b).

Incubation with *B. bifidum* S17/pMgapS6C and CB1954 resulted in efficient growth inhibition of HT-29/EGFP4 MCTS compared to the negative controls. The EGFP signal of MCTS treated with *B. bifidum* S17/pMgapS6C or CB1954 alone did not differ from untreated controls. However, fluorescence of MCTS treated with a combination with *B. bifidum* S17/pMgapS6C and CB1954 was reduced significantly and was comparable to that observed with staurosporine treatment (Fig. 5c). This indicates efficient growth inhibition of HT-29/EGFP4 MCTS upon treatment with the recombinant strain and the prodrug.

## Discussion

Delivery of therapeutic genes by anaerobic bacteria is an attractive alternative or supplementary strategy in cancer treatment [12–15]. The vast majority of studies on bacterial tumor targeting have been performed in mouse models. In the present study, 3D MCTS of the colorectal adenocarcinoma cell line HT-29 were evaluated as a model system to investigate bacterial tumor targeting in vitro.

HT-29 MCTS displayed (patho)physiologically relevant features of solid tumors. Size and organization of these MCTS into a proliferating outer zone and increasing hypoxia, apoptosis and necrosis towards the core are in line with previous reports [37, 38]. The presented data provides direct evidence for a selective localization of tumor targeting bacteria in hypoxic areas of 3D tumor spheroids. *B. bifidum* S17 derivatives expressing the fluorescent proteins GFP or mCherry were detected almost exclusively in hypoxic areas of MCTS. *S. typhimurium* YB1 localized preferentially to hypoxic areas of MCTS and appeared to proliferate in this niche (Fig. 2). Both strains are able to survive at high rates for at least 72 h in MCTS. By contrast, viable bacteria of both strains decreased significantly in controls, i.e. medium without MCTS. Slight differences in the viability under these conditions were noted with a more rapid decrease observed for *B. bifidum* S17 compared to *S. typhimurium* YB1. This may be due to the fact that bifidobacteria are obligate anaerobes to which presence of oxygen is detrimental. *S. typhimurium* strains are facultative anaerobes, which are able to grow without oxygen but prefer growth under aerobic conditions. The YB1 strain is genetically modified in a way that they are not able to grow in the presence of oxygen [36]. However, oxygen is not toxic to YB1 which maybe the reason for the better survival of YB1 under cell culture conditions.

*Bifidobacterium bifidum* S17 and *S. typhimurium* YB1 were found in slightly different locations of the MCTS. While *B. bifidum* S17 was detected almost exclusively in hypoxic areas of tumors, *S. typhimurium* YB1 also adhered to the outer surface. This might be due to differences in the labeling methods. *B. bifidum* S17 was detected by expression of fluorescent proteins. By contrast, *S. typhimurium* YB1 was labeled with a specific antibody. Whereas the former method detects only intact, metabolically active cells, antibodies stain all bacteria including dead or metabolically inactive cells. Thus, it is possible that the signal of *S. typhimurium* YB1 detected on the outside of MCTS represents non-replicative, inactive, or dead bacteria.

Nevertheless, *S. typhimurium* YB1 was shown to proliferate in MCTS at later time-points indicating that some bacteria, presumably those that reside in hypoxic areas, are viable. Similarly, expression of fluorescent proteins by *B. bifidum* S17 and germination of *C. sporogenes* spores in hypoxic areas of MCTS suggest metabolic activity. Moreover, germination of spores of strictly anaerobic *C. sporogenes* NCIMB 10696 was observed inside MCTS (Fig. 3). This suggests that oxygen levels inside MCTS are sufficiently low to support colonization of hypoxic areas of HT-29 MCTS by obligate anaerobic bacteria. Thus, the presented data confirms previous findings on selective colonization of solid tumors by these bacteria in animal models [16, 17, 25, 35, 36].

These findings indicate that recombinant, tumor targeting bacteria are likely to express therapeutic genes inside MCTS. To test the potential of MCTS for studies on therapeutic approaches, derivative of the HT-29 cell line stably transfected for constitutive EGFP expression was generated. Techniques employing fluorescent labels or read-outs are widely used for high-throughput drug discovery [47, 48] and EGFP-expressing HT-29 tumor spheroids have been generated previously for the high-throughput screening of pharmacological treatments [49]. Proliferation of entire spheroids was measured by EGFP fluorescence intensity using a microtiter plate reader. This is an easy, reliable and fast measurement. While flow cytometry would be a theoretical alternative providing quantitative information on the total number of cells in spheroids and the fluorescence intensity on the single cell level, it bears downsides that are related to the spheroid model. For example, spheroids need to be disintegrated to a single cell suspension prior to flow cytometry. In fact, several attempts to reproducibly and reliably obtain single cell suspension from HT-29 MCTS by enzymatic treatment with or without mechanical disruption failed (data not shown) probably due to the tight cell–cell adhesion of cells within spheroids. Staurosporine treatment of HT-29/EGFP4 MCTS resulted in a dose-dependent inhibition of the increase in EGFP fluorescence of MCTS (Additional file 3: Figure S2C) as reported for other cell lines [50] demonstrating the principal feasibility of HT-29/EGFP4 MCTS to test therapeutic strategies.

To test therapeutic approaches using bacterial gene delivery vectors, recombinant strains of *B. bifidum* S17 were generated expressing two of the most widely used PCEs: cytosine deaminase and chromate reductase. Both PCEs have been used successfully in bacterial tumor targeting studies in animals models [51, 52]. In MCTS, a combination of *B. bifidum* S17/pMgapS6C and CB1954 was able to inhibit tumor growth (Fig. 5c). By contrast, *B. bifidum* S17/pAO-S0_CD expressing cytosine deaminase in combination with the prodrug 5-FC had no effect on growth of HT-29 MCTS (Fig. 4a). Further experiments revealed that the clone of HT-29 cells used for experiments had an intrinsic resistance to the active compound 5-FU produced by the PCE (Fig. 4b). Similar observations have been published for this and other colon cancer cell lines [44, 45].

The vast majority of reports on bacterial tumor-targeting have been performed in mouse models and there are very few studies on bacterial tumor targeting using 3D in vitro models. To our knowledge, there is only one study in which tumor spheroids were used to investigate the therapeutic effect of a recombinant *S. typhimurium* strain expressing a therapeutic gene [53]. A microfluidic tumor model was used to analyze proliferation of different *E. coli* strains in [54]. This system consists of a reactor chamber with a diameter 100 µm connected to two outer channels perfused with oxygenated medium by pores of 4 µm allowing diffusion of nutrients and oxygen. Thus, it probably does not allow for the development of extensively hypoxic areas, which in MCTS start not until approx. 100 µm below the outer surface. Tumor cylindroids, which are derived from MCTS, have been used study tumor colonization by *S. typhimurium* [55–57]. Compared to MCTS, both cylindroids and microfluidics are technically by far more challenging, require significant additional equipment, and are thus not suitable for large scale or high throughput screenings.

## Conclusions

Collectively, our results demonstrate that MCTS generated from HT-29 cells are a suitable model system to study tumor targeting strategies using obligate anaerobic bacteria or their spores in vitro. A wide range of other tumor cell lines have been shown to form similar MCTS when cultured appropriately [37–39]. Moreover, MCTS can be grown in a high-throughput format [42] or developed further into more complex model systems offering the opportunity to analyze the molecular cross-talk between tumor cells and other cell types frequently found in tumors such as fibroblasts or immune cells [38, 58–60]. This may facilitate research on therapeutic approaches beyond PCE strategies, e.g. recombinant bacterial vectors expressing defined cytokines that modulate the anti-inflammatory phenotype of tumor-associated macrophages or activate infiltrating T- or NK-cells.

In conclusion, MCTS have great potential to facilitate preclinical investigation of bacterial tumor targeting. MCTS allow efficacy testing of existing bacterial vectors, the development of new therapeutic strategies involving bacterial gene delivery, or oncolytic effects of bacteria an in vitro setting before turning to time, cost, and labor intensive animal experimentation.

## Methods

### Bacterial strains and growth conditions

Bifidobacterial strains were grown in Lactobacilli MRS medium (Difco) supplemented with 0.5 g/L l-cysteine (MRSc) under anaerobic conditions (AnaeroGenTM, Oxoid) at 37 °C. For cultivation of *B. bifidum* S17 derivatives carrying plasmids pVG-mCherry, pVG-GFP, pAO-S0_CD [43, 61], or pMgapS0C, MRSc was supplemented with 100 µg/mL spectinomycin. *S. typhimurium* YB1 [36] was kindly provided by Prof. Jian-Dong Huang (University of Hong Kong) and was grown aerobically at 37 °C in DMEM cell culture medium supplemented with 50 µg/mL DAP and 25 µg/mL chloramphenicol. *Clostridium sporogenes* NCIMB 10696 (NCIMB Ltd., Aberdeen, GB) was cultivated anaerobically in 2YT medium containing 3 % (w/v) tryptone, 2 % (w/v) yeast extract, and 8.7 mM sodium thioglycolate.

For generation of *C. sporogenes* spores, 50 mL medium was inoculated and incubated for 2 weeks at 30 °C. Sporulation was monitored microscopically. Spores were harvested by centrifugation (30 min, 4 °C, 4500×*g*) and incubated for 30 min in 50 mL 80 % (v/v) ethanol to kill any remaining vegetative cells. The pellet was washed three times with 50 mL phosphate buffered solution (PBS) and suspended in 125 µL PBS. Spores were separated by density centrifugation using Percoll^®^. For this purpose 900 µL of filtrated 1.5 M NaCl was added to 8.1 mL of Percoll^®^ solution (Sigma-Aldrich Chemie GmbH, Munich, Germany). 9 mL of this stock solution were mixed with 1 mL of 0.15 M NaCl. A gradient was prepared by centrifugation using a swinging bucket rotor (1 h, room temperature, 4500×*g*). 125 µL of crude spore preparation was applied on top of the gradient followed by centrifugation (1 h, room temperature, 1800×*g*). The upper 9 mL of the Percoll^®^/NaCl gradient were removed and the spore sediment was resuspended in 5 mL of PBS followed by centrifugation (5 min, RT, 9600×*g*). The pellet was washed three times with PBS and resuspended in 1 mL PBS. Absence of vegetative cells was checked microscopically. The concentration of colony-forming units (CFU) of the spore preparation was determined by plating on 2YT agar and cultivation at 30 °C under anaerobic conditions.

### Cell culture and generation of solid 3D MCTS in vitro

HT-29 colorectal adenocarcinoma cells (ATCC^®^ HTB-38™) were routinely cultured in DMEM medium supplemented with 5 % (v/v) FCS, 1 % (v/v) non-essential amino acids, and 1 % (v/v) penicillin–streptomycin stock solution at 37 °C with 5 % CO_2_. For generation of MCTS, cells were seeded in Ultra-Low Attachment 96-well plates (Corning^®^ Costar^®^) at 1 × 10^5^ cells in 200 µL medium per well. Cells were grown for 14 days with medium changed every 2–3 days. MCTS with a diameter of 500–1000 µm were used for experiments.

To generate green fluorescent MCTS, HT-29 cells were transfected with pEGFP-N3 (Clontech Laboratories, Inc., USA) and a stable clone HT-29/EGFP4 was selected as described in Additional file 2: Materials S2. HT-29/EGFP4 MCTS were generated using DMEM medium without phenol red to avoid interference with EGFP fluorescence measurements. Growth of HT-29/EGFP4 MCTS was monitored by quantification of EGFP fluorescence as an indirect measurement of cell proliferation and MCTS size. Fluorescence intensity of HT-29/EGFP4 MCTS was measured directly in tissue culture plates using a Tecan Infinite^®^ M200 multimode reader (Tecan Group Ltd., Mannedorf, Switzerland) with excitation at 477 nm and emission at 507 nm.

### Bacterial tumor targeting assays

For survival studies in MCTS, bifidobacteria were inoculated in 10 mL MRSc supplemented with antibiotics as appropriate. *S. typhimurium* YB1 was inoculated in 10 mL DMEM containing 50 µg/mL DAP and 25 µg/mL chloramphenicol. Bacteria were grown anaerobically (bifidobacteria) or aerobically (*S. typhimurium* YB1) at 37 °C over night (o/N; i.e. for 16 h) to stationary growth phase. At this stage, an optical density at 600 nm (OD_600_) of 1 equals approximately 1 × 10^8^ (bifidobacteria) or 1 × 10^9^ (*S. typhimurium* YB1) CFU/mL. Bacteria were washed once and resuspended in PBS to a final OD_600_ of 0.1 (bifidobacteria) or 0.01 (*S. typhimurium* YB1). The *C. sporogenes* spore preparation was adjusted to 5 × 10^5^ CFU/mL.

Prior to inoculation, single MCTS in individual wells were washed once in DMEM and 180 µL of DMEM supplemented with 60 mM HEPES buffer (without FCS or antibiotics) were added. Aliquots of 20 µL of the bacterial suspensions or spore stocks were added to individual wells resulting in an inoculum of 2 × 10^7^ CFU or 1 × 10^3^ spores per MCTS. At the indicated time-points after infection, six MCTS infected with the same strain were combined. Homogenous suspensions of tumor cells were prepared by passing through a syringe (0.4 mm) 5 times with vortexing between homogenization steps. Bacterial numbers were determined as CFU/mL in the MCTS lysates by plating serial ten-fold dilutions in PBS on agar plates (bifidobacteria: MRSc agar; *S. typhimurium* YB1: LB agar containing 50 µg/mL DAP after growth under standard conditions. For assays with *C. sporogenes* spores, MCTS were infected and incubated for 7 days under cell culture conditions. At this stage tumors were examined by fluorescence microscopy.

For experiments with recombinant bifidobacteria expressing PCEs, MCTS were incubated with 2 × 10^7^ CFU. Medium was changed after 72 h of incubation and fresh prodrug was added to MCTS.

### Microscopic examination of MCTS

Individual MCTS incubated with bacteria or spores for the indicated time were embedded in a drop of TissueTek^®^ O.C.T Compound (Sakura) in a Leica CM3050S cryostat and immediately used to prepare cryosections (thickness 10–14 µm), which were then dried on Superfrost™ Plus microscope slides (Fisher Scientific) and fixed with 4 % paraformaldehyde/PBS.

Fixed MCTS were stained with hematoxylin/eosin (H&E). Hoechst (Life Technologies) was used at 300 nM to stain cell nuclei and cytoskeletal actin was stained using Alexa Fluor^®^ 488 Phalloidin (Invitrogen). Hypoxyprobe-Red549 Kit (Hypoxyprobe) was used to stain for hypoxic areas and apoptotic cells were stained by TUNEL assay (DNA Fragmentation Imaging Kit, Roche). Proliferating cells were visualized by Ki-67 staining using a mouse monoclonal antibody (Cat# MA1-80199, Thermo Scientific) and goat α-mouse IgG Alexa Fluor^®^ 555 secondary antibody (Life Technologies). *S. typhimurium* YB1 was stained with a rabbit polyclonal *Salmonella* antiserum (Cat# PA1-7244, Thermo Scientific) and goat α-rabbit Alexa Fluor^®^ 488 or 555 secondary antibodies (Life Technologies). Clostridial spores were stained with polyclonal, FITC-conjugated Clostridia species antibodies (Cat# PA1-73169, Thermo Scientific). In a mix of vegetative *C. sporogenes* cells only spores were stained by the antibody while DNA of vegetative cells was stained by Hoechst demonstrating specificity of the antibody for spores.

Microscopic analysis was performed with an Axio Observer.Z1 microscope (Zeiss) using Zen software. The following filter sets were used for fluorescence microscopy: 49 (excitation at 365 ± 10 nm, emission at 445 ± 50 nm) for Hoechst, 38HE (excitation at 470 ± 40 nm, emission at 525 ± 50 nm) for GFP, TUNEL, FITC and Alexa Fluor^®^ 488, and AF (excitation at 545 ± 30 nm, emission at 610 ± 75 nm) for mCherry, Hypoxyprobe-Red549, and Alexa Fluor^®^ 555.

### Cloning of PCE-expressing *B. bifidum* S17 strains

*Bifidobacterium bifidum* S17/pAO-S0_CD expressing a secreted cytosine deaminase has been described previously [43]. A similar construct was devised to generate a recombinant *B. bifidum* S17 strain expressing a secreted chromate reductase. For this purpose, the *chrR6* gene encoding an artificial nitroreductase created by mutation of the *E. coliyieF* gene, which shows faster enzyme kinetics than its native form, was used [62]. The *chrR6* gene was codon-optimized for *B. bifidum* S17 and fused in silico to the coding sequence of S6, a signal peptide of *B. bifidum* S17 for efficient protein secretion [43]. The codon-optimised fusion was synthesised by a commercial provider (Eurofins Genomics GmbH; Germany) and amplified by PCR using primers S6F (3′-GGCCTCGAGATGAAATCACTGATGAAAAAGGTTTTCGC-5′) and ChrR (3′-CCCAAGCTTTTAAATTTTCACGCGTTG-5′) to introduce appropriate restriction sites (underlined in primer sequences). The obtained PCR product was digested with restriction enzymes XhoI and HindIII and ligated to the 4423 bp fragment of XhoI/HindIII digested pMDY23-P_*gap*_ [63] yielding pMgapS6C, which encodes an exact transcriptional fusion of the S6-*chrR6* sequence to the *gap* promoter of *B. bifidum* S17 for strong constitutive expression [64].

Following transformation into *E. coli*, plasmids of spectinomycin resistant colonies were checked for correct inserts by PCR. Plasmids of positive clones were verified by restriction analysis and Sanger sequencing. A plasmid with correct sequences and a mutation-free insert was termed pMgapS6C. This plasmid and the empty vector control (pMDY23-P_*gap*_) were transformed into *B. bifidum* S17 as described elsewhere [65]. Chromate reductase activity was measured in culture supernatants of recombinant bifidobacteria grown for 16 h in Reinforced Clostridium Medium at 37 °C under anaerobic conditions using a previously described enzymatic assay [66].

## Additional files

10.1186/s12934-015-0383-5 Characterisation of HT-29 MCTS.
10.1186/s12934-015-0383-5 Generation of HT-29/GFP4 cells.
10.1186/s12934-015-0383-5 Characterization of HT-29 and HT-29/EGFP4 cells.

## Abbreviations

3D: three-dimensional
MCTS: multicellular tumor spheroids
PCE: prodrug-converting enzyme
5-FC: 5-fluorocytosine
5-FU: 5-fluorouracil
