# Supplementary material for: Three-dimensional tumor spheroids for in vitro analysis of bacteria as gene delivery vectors in tumor therapy
Source: Microb Cell Fact. 2015 Dec 12;14:199. doi: 10.1186/s12934-015-0383-5 (PMC4676896; doi:10.1186/s12934-015-0383-5)
Supplement: Supplementary file 1 — 10.1186/s12934-015-0383-5 Characterisation of HT-29 MCTS. [file 12934_2015_383_MOESM1_ESM.pdf]

Figure S1: Characterisation of HT-29 MCTS.

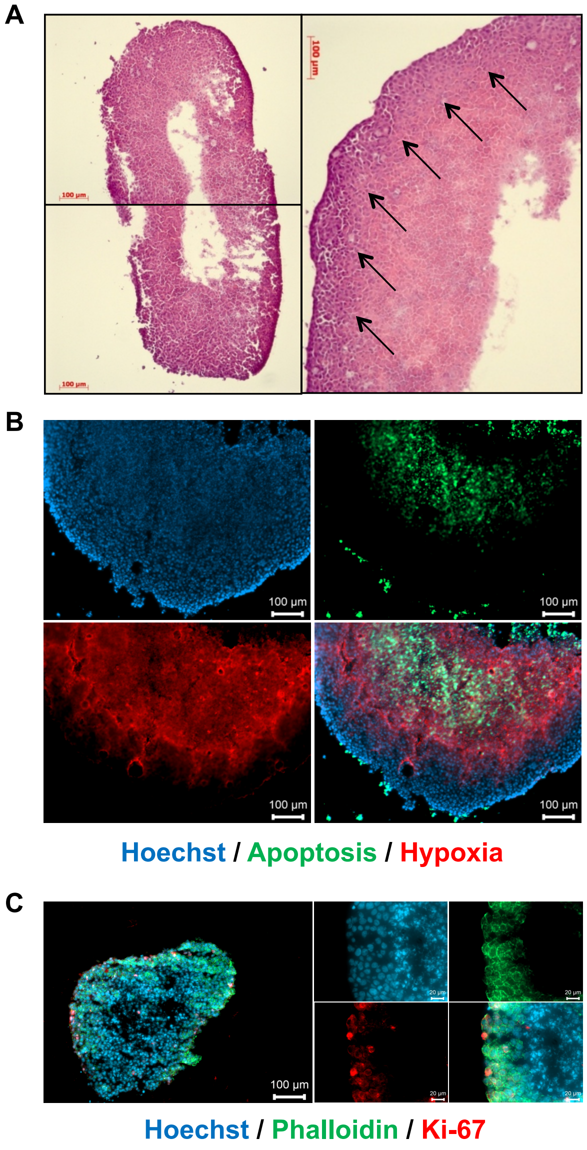

Figure S1: Characterisation of HT-29 MCTS. (A) H&E stained histological sections of representative MCTS formed by HT-29 cells cultured for 14 days. The two panels to the right are a composite of two adjacent fields of view. The image in the left panel is a zoom-in on a part of the same section. Arrows indicate areas of cells with intact nuclei stained with hematoxylin (purple) surrounding eosin-stained areas (pink). (B) and (C) Fluorescence microscopy of HT-29 MCTS cryosections. Nuclei were stained with Hoechst (blue). Staining in (B): hypoxia (pimonidazole and α-pimonidazole antibody, red) and apoptosis (DNA strand

breaks by TUNEL, green). Staining in (C): proliferating cells (α-Ki-67 antibody, red) and actin cytoskeleton (Alexa Fluor® 488 Phalloidin, green). The results clearly demonstrate that HT-29 MCTS display the typical tissue architecture of solid tumours with an outer zone of proliferating cells with intact nuclei and defined actin cytoskeleton and increasing hypoxia, apoptosis, necrosis and loss of structured cytoskeleton towards the core. Images were acquired with a Zeiss Axio Observer.Z1 microscope using a 10× (all panels in A and B and left panel in C, scale bars 100 µm), or a 40× objective (small panels in C, scale bars: 20 µm).
