# Supplementary material for: Three-dimensional tumor spheroids for in vitro analysis of bacteria as gene delivery vectors in tumor therapy
Source: Microb Cell Fact. 2015 Dec 12;14:199. doi: 10.1186/s12934-015-0383-5 (PMC4676896; doi:10.1186/s12934-015-0383-5)
Supplement: Supplementary file 2 — 10.1186/s12934-015-0383-5 Generation of HT-29/GFP4 cells. [file 12934_2015_383_MOESM2_ESM.docx]

**Materials S2: Generation of HT-29/GFP4 cells**

HT-29 cells were used to generate a stable EGFP-expressing cell line by transfection with pEGFP-N3 (Clontech Laboratories, Inc., USA). HT-29 cells were seeded in 1 mL of in DMEM medium supplemented with 5 % (v/v) FCS, 1 % (v/v) non- essential amino acids, and 1 % (v/v) penicillin-streptomycin in 24 well tissue culture plates at 1×10^5^ cells/well and grown for 48 h under standard conditions. Prior to transfection, medium was changed to standard medium without FCS and HT-29 cells were transfected with 0.8 µg plasmid DNA and 3 µL transfection reagent according to the manufacturers instructions. After 4 h of cultivation in the presence of transfection reagent and plasmid DNA, medium was changed to DMEM supplemented with 20 % (v/v) FCS and 1 % (v/v) non-essential amino acids. After 24 h of cultivation, geniticin was added to a final concentration of 0.5 mg/mL and cells were further grown on the presence of geniticin with medium changes 2-3 times per week until single small colonies became visible. The colonies were picked and subcultured separately until a sufficient number of cells for FACS analysis, experiments, and cryostocks were available. A total of four stable geneticin-resistant clones expressing EGFP were obtained. All four clones were analyzed for growth and EGFP fluorescence. Of the four clones obtained, clone 4 (HT-29/EGFP4) was selected for further experiments based on high fluorescence intensity, absence of EGFP-negative cells (Supplementary Figure S5), and indistinguishable growth characteristics compared to the parental HT-29 cell line (data not shown).
