# Supplementary material for: Three-dimensional tumor spheroids for in vitro analysis of bacteria as gene delivery vectors in tumor therapy
Source: Microb Cell Fact. 2015 Dec 12;14:199. doi: 10.1186/s12934-015-0383-5 (PMC4676896; doi:10.1186/s12934-015-0383-5)
Supplement: Supplementary file 3 — 10.1186/s12934-015-0383-5 Characterization of HT-29 and HT-29/EGFP4 cells. [file 12934_2015_383_MOESM3_ESM.pdf]

**Figure S2: Characterization of HT-29 and HT-29/EGFP4 cells.**

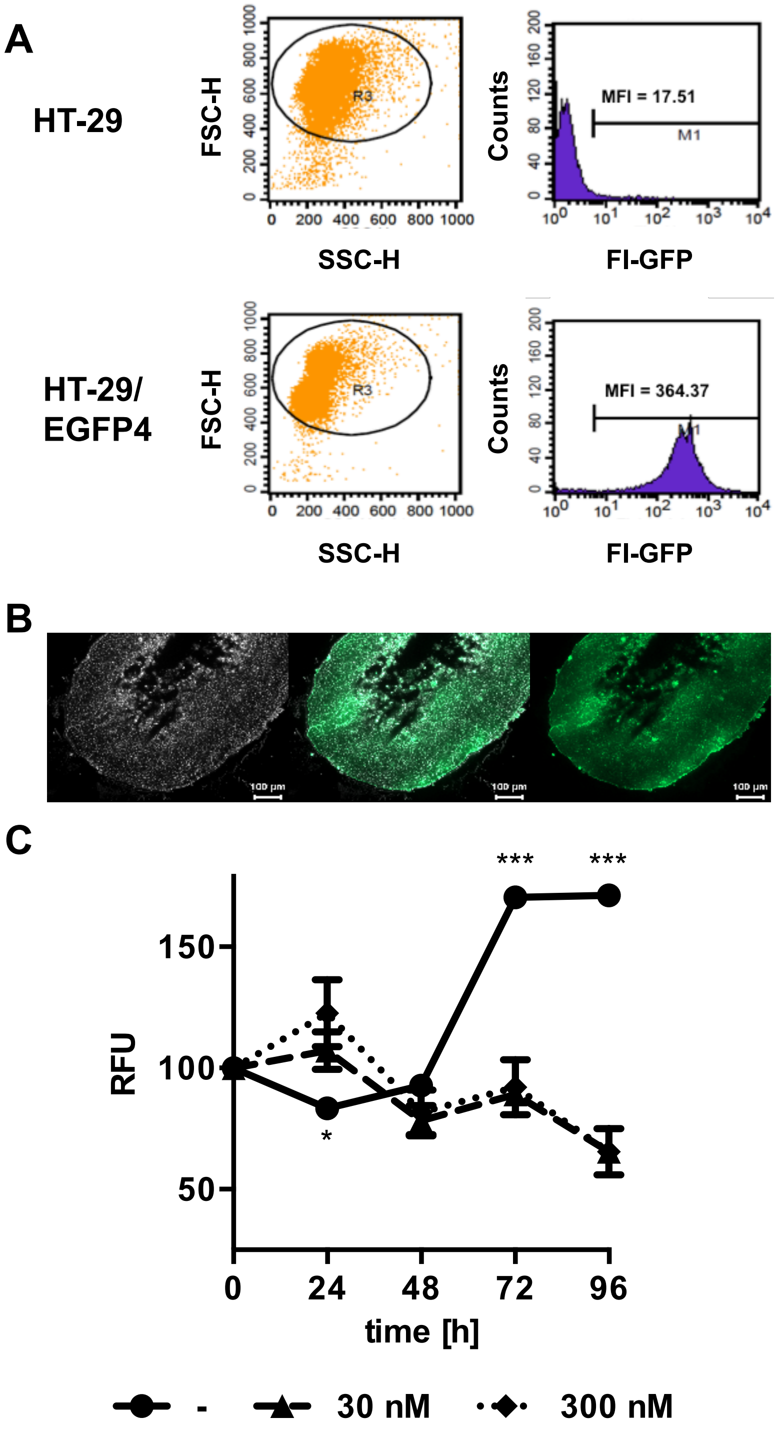


**Figure S2: Characterization of HT-29 and HT-29/EGFP4 cells.** (A) Parental HT-29 and HT-29/EGFP4 cells were cultivated in 75 cm$^2$ tissue culture flasks to approx. 80 % confluence and analysed by flow cytometry for size (forward scatter; FSC-H) and granularity (side scatter, SSC-H; left panels). Healthy, live cells in gate R3 were used for analysis of

green fluorescence in the FL1 channel (FI-GFP; right panels) and mean fluorescence intensity (MFI) of the EGFP positive cells (M1). (B) Fluorescence microscopy of cryosections of HT-29/EGFP4 MCTS (upper panel: brightfield; lower panel: GFP channel; middle: merge). Images were acquired with a Zeiss Axio Observer.Z1 microscope using a 10× objective (scale bars: 100 µm). (C) Fluorescence of HT-29/EGFP4 MCTS during growth in the presence or absence of staurosporine (30 or 300 nM). Fluorescence intensities were normalized to give 100% for each MCTS at the start of the experiment (t = 0 h). Values are relative fluorescent units (RFU) per MCTS and are mean ± SEM of three MCTS per condition. Results are from one representative of three independent experiments. Statistical analysis was performed using two-way ANOVA with Bonferroni post-test correction for multiple comparisons. Astersisk indicate levels of statistical significance of untreated compared to both treatment groups (*:P < 0.05; ***: P < 0.001, all other comparisons not significant).
